# Mitochondrial damage and radiation carcinogenesis

**DOI:** 10.1093/jrr/rrt170

**Published:** 2014-03

**Authors:** Tom K. Hei

**Affiliations:** 1 Center for Radiological Research, Department of Radiation Oncology, College of Physicians and Surgeons, Columbia University Medical Center, New York, NY 10032 USA; 2 Department of Environmental Health Sciences, Mailman School of Public Health, Columbia University Medical Center, New York, NY 10032, USA

**Keywords:** space radiation, tumor suppressor, mitochondrial fusion and fission, genomic instability

## Abstract

Although radiation is a well-established human carcinogen, the mechanism of how radiation induces cancer is not clear. High linear energy transfer (LET) particles such as those used in radiotherapy and found in the natural radiation environment in space are potent clastogens that induce chromosomal breakages and present a potential mechanism for the loss of tumor suppressor genes. Indeed, using an immortalized human bronchial epithelial cell line, we show previously that high LET radiation, including alpha and HZE particles, induces a stepwise neoplastic transformation of these cells and a consistent downregulation of the tumor suppressor *TGFBI* gene*.* Furthermore, there is recent evidence that targeted cytoplasmic irradiation induces changes in mitochondrial morphology. Mitochondria are the energy center of a cell and normal mitochondria are highly dynamic organelles that move along microtubules or microfilaments and continuously fuse and divide in healthy cells. A balance between mitochondrial fusion and fission is essential to maintain normal mitochondrial function. In contrast, irradiated cells show shortened and fragmented mitochondria together with a reduction in mitochondrial functions. These included reduced cytochrome C-oxidase as well as succinate dehydrogenase activities when compared with non-irradiated controls, suggestive of reduced respiratory metabolism. Furthermore, irradiated airway epithelial cells showed an increase in mitochondrial superoxide production that was quenched by the radical scavenger, dimethyl sulfoxide. This acute mitochondrial response caused by cytoplasmic irradiation may result in the release of several stress mediators, which are necessary for mitochondria to preserve cellular homeostasis. The observation that progeny of mammalian cells that are irradiation through the cytoplasm show evidence of genomic instability highlights the functional role of mitochondrial damage in radiation carcinogenesis.

